# Systemic Tumors Can Cause Molecular Changes in the Hippocampus That May Have an Impact on Behavior after Chronic Social Stress

**DOI:** 10.3390/neurosci5020014

**Published:** 2024-06-06

**Authors:** Olatz Goñi-Balentziaga, Alina Díez-Solinska, Garikoitz Beitia-Oyarzabal, Maider Muñoz-Culla, Garikoitz Azkona, Oscar Vegas

**Affiliations:** 1 Department of Clinical and Health Psychology, and Research Methods, School of Psychology, University of the Basque Country (UPV/EHU), 20018 Donostia-San Sebastian, Spain; olatz.goni@ehu.eus; 2 Department of Basic Psychological Processes and Their Development, University of the Basque Country (UPV/EHU), 20018 Donostia-San Sebastian, Spain; alinaisabel.diez@ehu.eus (A.D.-S.); garikoitz.beitia@ehu.eus (G.B.-O.); maider.munoz@ehu.eus (M.M.-C.); 3 Biogipuzkoa Health Research Institute, 20014 Donostia-San Sebastian, Spain

**Keywords:** chronic social stress, monoamines, kynurenine pathway, tumor development, hippocampus

## Abstract

Evidence indicates that chronic social stress plays a significant role in the development of cancer and depression. Although their association is recognized, the precise physiological mechanism remains unknown. In our previous work, we observed that OF1 males subjected to chronic social defiance exhibited anhedonia, and those who developed tumors in the lung showed anxiety-associated behaviors. In this study, we observed that tumor-bearing OF1 mice presented higher levels of 3-HK, and this increase may be due to IDO. No differences in hippocampal catecholamine levels were observed. Our results suggest that a systemic tumor can induce molecular changes in the hippocampal kynurenine pathway that may impact behavior.

## 1. Introduction

In social animals, lifelong health and well-being depend on social networks and social support [1]. However, a lack of social interaction or unsatisfactory social interactions can be a source of stress. The physiological stress response involves the co-activation of the sympathetic–adrenal–medullary (SAM) and hypothalamic–pituitary–adrenal (HPA) axes after perceiving a threat. Adrenaline (A), noradrenaline (NA), and glucocorticoids (CORT) are released by the suprarenal gland, facilitating rapid adaptation [2,3]. The adaptive responses observed in physiological and behavioral reactions to acute stress contrast with the potential adverse effects of chronic stress. Prolonged exposure to social stressors can increase vulnerability to chronic diseases, including depression [4,5] and cancer [6].

Numerous lines of evidence indicate that chronic psychosocial stress plays a significant role in cancer development, contributing to higher mortality rates across various cancer types [7,8]. The elevated prevalence of depressive disorders among cancer patients suggests a connection between the two conditions [9,10] not solely explained by the psychosocial stress associated with cancer itself [11,12].

Although the association between stress, depression, and cancer is recognized, the precise physiological mechanism remains unknown. A recently proposed hypothesis revolves around inflammation resulting from stress-induced neuroendocrine changes and the presence of the tumor itself [13,14,15,16].

The utilization of animal models, particularly chronic defeat stress (CDS), has proven invaluable in exploring individual variations in behavioral and physiological responses to chronic social stress. At the behavioral level, chronic exposure to the resident–intruder paradigm induces anxiety-like and depressive-like behavior in male mice [17,18,19,20,21,22]. Both systemic and encephalic immunological changes often accompany these behavioral changes [23,24,25,26,27,28]. Several studies have successfully reversed the behavioral phenotype by stimulating the innate immune system [29,30].

On the other hand, chronically elevated inflammatory cytokine levels result in changes in the monoamine system [31,32]. The monoamine hypothesis proposes that diminished dopamine (DA), NA, and serotonin (5-hydroxytryptamine, 5-HT) signal pathways may contribute to depression [33]. Catecholamine biosynthesis begins with the conversion of phenylalanine (Phe) to tyrosine (Tyr). DA is synthesized from Tyr in dopaminergic neurons and NA from DA in noradrenergic neurons. Various enzymes metabolized DA to 3,4-dihydroxyphenylacetic acid (DOPAC) and NA to 3-methoxy-4-hydroxyphenylglycol (MHPG) [34], while 5-HT is synthesized from tryptophan (Tryp) and metabolized to 5-hydroxyindoleacetic acid (5-HIAA). Most Tryp enters the kynurenine pathway. Kynurenine (Kyn), produced from Tryp, is converted to kynurenic acid (Kyna) or 3-Hydroxykynurenine (3-HK), the latter converting to quinolinic acid, which affects both the monoaminergic and glutamatergic neurons [35]. Chronic stress can shift metabolism away from 5-HT production towards the kynurenine pathway [36]. Male OF1 mice inoculated with B16F10 melanoma tumor cells showed higher immobility in the tail suspension test, decreased dopaminergic activity in the striatum, and 5HT turnover in the prefrontal cortex [37]. However, the monoamine levels of these mice in the hippocampus were not analyzed.

In our previous work, we observed that adult male OF1 mice subjected to CSD exhibited higher levels of plasmatic CORT, anhedonia, and increased spleen weight. Those mice that developed tumors exhibited anxiety-associated behaviors independent of stress, but chronically defeated mice had more tumor foci than non-stressed mice [38]. In this current work, we aimed to analyze the levels of monoamines and the gene expression of the *tumor necrosis factor (TNF)-alpha* and the *interleukin (IL)-6* cytokines in the hippocampus of these same mice. This brain area is particularly susceptible to the action of CORT and is affected in patients suffering from depression. The study of the possible molecular mechanisms of the effect of stress on the immune system and tumor development may open up new perspectives for understanding the neurobiological basis of this relationship, which is not yet known. 

## 2. Materials and Methods

Six-week-old OF1 outbred male mice (Charles River Laboratories, Evreux, France) were housed in transparent plastic cages measuring 24.5 × 24.5 × 15 cm. Food and water were available ad libitum. The holding room was maintained at a constant temperature of 22 ± 2 °C with a relative humidity level of 70% and a reverse 12 h light/dark cycle (white lights on from 19:00 to 07:00) to enable the testing of these nocturnal animals during their active phase (1 h after the beginning of the dark cycle). All experimental procedures were conducted under dim red lighting in a room adjacent to the holding facility. All procedures involving mice were performed following the European Directive (2010/63/EU) and were approved by the Animal Welfare Ethics Committee of the University of the Basque Country (CEEA-UPV/EHU; M20/2018/090) and the Gipuzkoa Provincial Council (PRO-AE-SS-062).

The day before starting the CSD, animals were randomly allocated to two groups, one with B16F10 melanoma cells (*n* = 51) and another not inoculated (*n* = 51). Anesthetized mice (isoflurane 2%) were inoculated with 5 × 104 viable B16F10 cells in 0.1 mL medium via the lateral tail vein using a 30.5 gauge needle after the tail had been heated with a thermal pad. All subjects received the full 0.1 mL dose in one injection. Each group was further divided into two subgroups, resulting in four experimental groups, namely stressed–tumor (*n* = 37), stressed–non-tumor (*n* = 14), non-stressed–tumor (*n* = 36), and non-stressed–non-tumor (*n* = 15). Animals in the stressed group were exposed to the sensory contact social stress model based on the resident–intruder paradigm. Each experimental subject was exposed to two 5 min daily interactions, followed by another 5 min non-social interactions with previously selected and trained highly aggressive resident mice. The stress period lasted for 18 days, and non-stressed mice were housed individually during this period. Over the next three days, animals underwent the sucrose preference test (SPT) and the forced swim test (FST) to analyze possible depressive-like behaviors and the open field test (OFT) to analyze locomotor activity (for more details, see [38]). On day 21, immediately after completion of the last behavioral test, the animals were sacrificed, and the hippocampi were removed. Tumor development was confirmed by incubating the lungs in Bouin’s solution for several days. The upper lobe of the left lung was then dissected, and the number of metastatic foci was counted using an Olympus SZ30 zoom stereomicroscope (Olympus, Tokyo, Japan).

We followed the same protocols previously described for our laboratory to determine monoamines and their metabolites by high-performance liquid chromatography (HPLC) [39] and mRNA gene expression by real-time PCR [39,40]. Briefly, total RNA was isolated using the NucleoSpin RNA Plus kit (Macherey Nagel, Düren, Germany). RNA concentrations were determined by spectrophotometric analysis at 260 nm (Synergy HT, BioTek Instruments, Inc., Winooski, VT, USA). Total RNA was then reverse transcribed using the PrimeScript RT reagent kit (Takara Bio Inc., Madrid, Spain). The resulting cDNA was quantified by SYBR Green-based (SYBR^®^Premix Ex TaqTM, Takara Bio Inc., Madrid, Spain) real-time PCR, and the formation of PCR products was monitored using the 7500 Real-Time PCR System (Applied Biosystems, Madrid, Spain). Both hypoxanthine phosphoribosyl transferase (HPRT) and glyceraldehyde-6-phosphate dehydrogenase (GAPDH) were used as reference genes. Primer sequences were designed using Primer Express Software v3.0 (Applied Biosystems, Madrid, Spain) and obtained from Applied Biosystems (Supplementary Table S1). Relative gene expression was determined using the 2-DΔt method.

Statistical analyses were performed using the GraphPad Prism software (9.0, GraphPad Software, Inc., La Jolla, CA, USA). Outlier values were identified using the ROUT method and removed from the analysis. Variables were analyzed using two-way ANOVA, and specific comparisons were analyzed with a post hoc Bonferroni test. Values of *p* < 0.05 were considered statistically significant. Cohen’s d test for the effect size was performed to estimate the strength of the effects between two groups (d values > 0.8 are considered large effects, values between 0.5 and 0.8 are considered moderate effects, and values < 0.5 are considered small effects). The results are described following the ARRIVE guidelines [41].

## 3. Results

**Figure 1:**
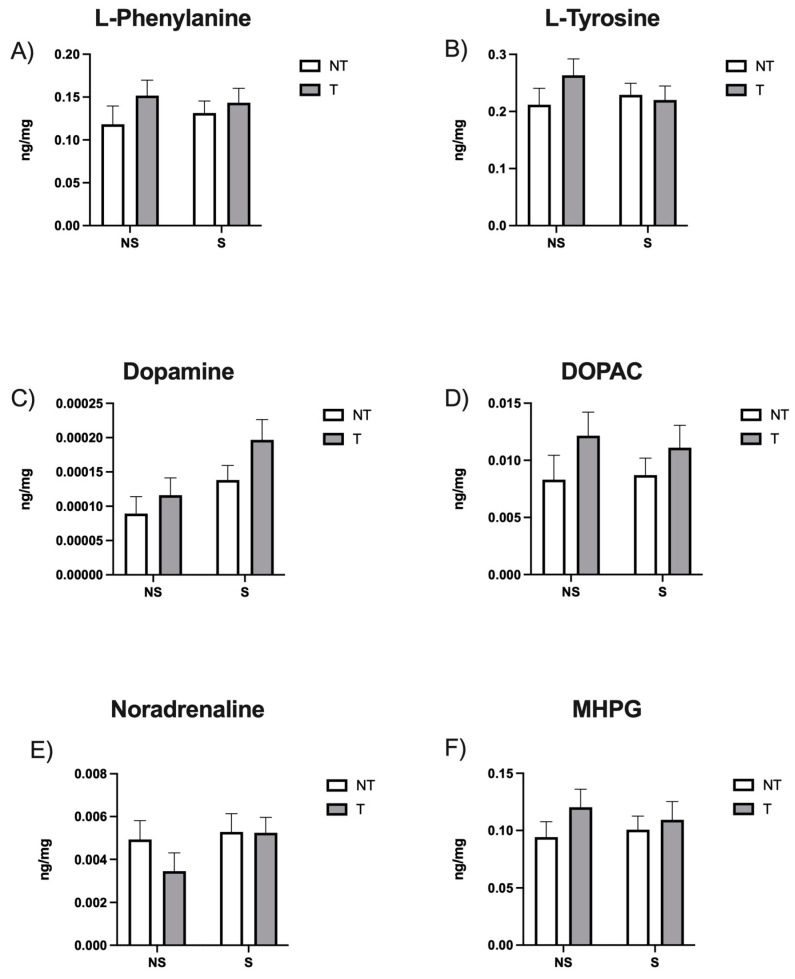
Hippocampal levels of (**A**) L-phenylalanine, (**B**) L-tyrosine, (**C**) Dopamine, (**D**) 3,4-Dihydroxyphenylacetic Acid (DOPAC), (**E**) Noradrenaline, and (**F**) 3-methoxy-4-hydroxyphenylglycol (MHPG) in ng/mg. Data are expressed as mean ± SEM. Factors: no stress (NS), stress (S), no tumor (NT), and tumor (T).

Our HPLC results indicate that there were no significant differences in hippocampal catecholamine levels among the groups (Figure 1).

**Figure 2:**
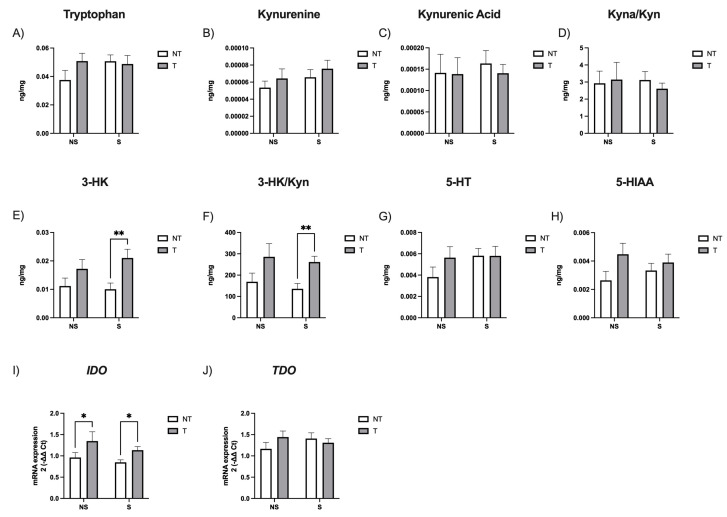
Hippocampal levels of (**A**) tryptophan, (**B**) kynurenine, (**C**) kynurenic acid, (**D**) Kyna/Kyn ratio, (**E**) 3-HK, (**F**) 3-HK/Kyn ratio, (**G**) 5-HT, (**H**) 5-HIAA and (**I**) *IDO* and (**J**) *TDO* mRNA expression. Data are expressed as mean ± SEM. Factors: no stress (NS), stress (S), no tumor (NT), and tumor (T). * *p* < 0.05, ** *p* < 0.001.

In the indolamine pathway, we observed no differences in the levels of Tryp, Kyn, and Kyna, nor the Kyna/Kyn ratio (Figure 2A–D). However, there was a significant difference in the tumor factor (*F*_(1,97)_ = 8.22, *p* = 0.005), but not in the stress factor (*F*_(1,97)_ = 0.002, *p* = 0.9), related to 3-HK levels. Post hoc analysis revealed that tumor-bearing stressed mice showed higher 3-HK levels than tumor-free stressed mice (*p* = 0.002, *d* = 0.80; Figure 2E). Similar results were obtained for the 3-HK/Kyn ratio (tumor: *F*_(1,77)_ = 10.70, *p* = 0.001; stress: *F*_(1,77)_ = 0.59, *p* = 0.4), with tumor-bearing stressed mice showing higher levels than tumor-free stressed mice (*p* = 0.002, *d* = 0.98; Figure 2F). No differences were observed in 5-HT and 5-HIAA levels (Figure 2G,H). 

We also analyzed *indoleamine-2,3-dioxygenase (IDO)* and *tryptophan 2,3-dioxygenase (TDO)* mRNA expression, the two main enzymes regulating the first and rate-limiting step of the Kyn pathway. Our results showed significant differences in IDO mRNA among groups for the tumor factor (*F*_(1,87)_ = 9.3, *p* = 0.002), but not for the stress factor (*F*_(1,87)_ = 2.22, *p* = 0.14). Tumor-bearing non-stressed mice expressed more IDO than non-tumor non-stressed mice (*p* = 0.04, *d* = 0.83), and tumor-bearing stressed mice than non-tumor stressed mice (*p* = 0.015, *d* = 0.61; Figure 2I). No difference was observed in TDO expression (Figure 2J).

**Figure 3:**
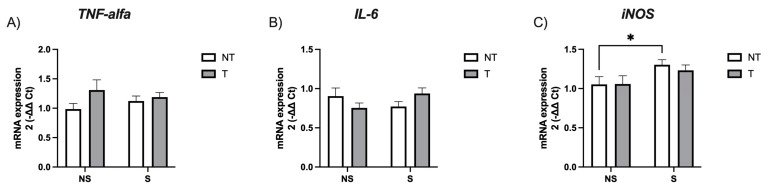
Hippocampal mRNA expression of (**A**) *TNF-alpha*, (**B**) *IL-6* and (**C**) *iNOS*. Data are expressed as mean ± SEM. Factors: no stress (NS), stress (S), no tumor (NT), and tumor (T). * *p* < 0.05.

We analyzed the mRNA expression of *TNF-alpha, IL-6*, and the inducible isoform of nitric oxide synthase (*iNOS*). Our results indicate no differences in cytokine levels, but a significant difference in *iNOS* expression in the stress factor (*F*_(1,96)_ = 5.69, *p* = 0.019) but not the tumor factor (*F*_(1,96)_ = 0.14, *p* = 0.7). Non-tumor-stressed mice showed significantly more expression than non-tumor non-stressed mice (*p* = 0.04, *d* = 0.63) (Figure 3).

## 4. Discussion

In this study, we analyzed monoamine levels in the hippocampus, as this is a brain region affected by chronic stress and involved in depressive-like symptoms. Concerning catecholamines, our results are consistent with previous work [35,36,37], suggesting that neither CSD nor tumors [38] induced changes in the levels of neurotransmitters, their precursors, or their metabolites.

Regarding the indolamine pathway, our results suggest that tumors, but not chronic stress, affect indolamine levels. In this regard, a previous paper reported that CSD induced an increase in Kyn and 3-HK with no changes in Tryp and 5-HT levels in the hippocampus of the stressed mice [42]. However, other studies reported no changes in 5-HT in the hippocampus of stressed mice [43] and tumor-bearing mice [38]. Overall, our results suggest that the systemic tumor induces changes in the tryptophan metabolism pathway, shifting it to the synthesis of quinolinic acid whose precursor is 3-HK. It has been described that the neurotoxic quinolinic acid affects the function of monoaminergic and glutamatergic neurons and can be involved in anxiety and depression [35,44]. Moreover, an increase in brain 3-HK has been linked to depression [45]. Thus, the increase in 3-HK could be behind the depressive-like phenotype observed in these animals.

This shift may be related to IDO, as its gene expression was increased in tumor-bearing animals. Although our data are at the RNA level, our results align with the prevailing theory that suggests that IDO plays an important role in the development of tumors, due to its immunosuppressive action in the host [46]. In our experiment, IDO upregulation does not appear to be mediated by IL-6 or TNF-alpha, but we cannot rule out the involvement of other pro-inflammatory cytokines or other mechanisms.

We analyzed the mRNA expression of the TNF-alpha and the IL-6 because IDO upregulation may be due to proinflammatory cytokine action [47], and higher plasma levels of these two proinflammatory cytokines have been reported in depressed subjects compared to controls [48]. However, we did not find differences between non-stressed and stressed mice in their mRNA expression. These results are inconsistent with previously reported data [25,37]. Finally, we observed higher iNOS expression in stressed mice, which has been related to depression- and anxiety-like behaviors [49].

It should be noted that few studies have simultaneously examined tumor development and analyzed monoamine levels in the brain; therefore, this study will certainly contribute an interesting perspective to the study of the possible mechanisms underlying the relationship between stress, cancer, and depression. However, there are some limitations to our study. We only analyzed one brain area in males. Therefore, we believe that in future projects we should carry out a more global analysis of the brain in mice of both sexes. Moreover, we should further analyze all the players involved in the tryptophan pathway to understand the specific molecular mechanisms underlying the depressive behavior.

## 5. Conclusions

We have previously published that CSD induced anhedonia and an increased number of tumor foci in the lung [38]. In this work, our results suggest that a systemic tumor can induce molecular changes in the hippocampal kynurenine pathway. Specifically, it increases 3-HK levels, but this effect is more pronounced in stressed mice and may be mediated by IDO. In conclusion, our results suggest that systemic tumors can produce molecular changes in the central nervous system that may impact behavior.

## Data Availability

Data from the study will be available upon reasonable request to the corresponding author.

## Supplementary Materials

The following supporting information can be downloaded at: https://www.mdpi.com/article/10.3390/neurosci5020014/s1, Table S1: PCR Primer specification.

## Abbreviations

3-Hydroxykynurenine (3-HK), 3-methoxy-4-hydroxyphenylglycol (MHPG), 3,4-Dihydroxyphenylacetic Acid (DOPAC), 5-hydroxytryptamine (5-HT), 5-Hydroxyindoleacetic Acid (5-HIAA), adrenaline (A), dopamine (DA), indoleamine-2,3-dioxygenase (IDO), inducible isoform of nitric oxide synthase (*iNOS*), interleukin (IL)-6, glucocorticoids (CORT), kynurenine (Kyn), kynurenic acid (Kyna), noradrenaline (NA), phenylalanine (Phe), tyrosine (Tyr), tryptophan (Tryp), tryptophan 2,3-dioxygenase (TDO), tumor necrosis factor (TNF)-alpha.
