# Supplementary material for: Systemic Tumors Can Cause Molecular Changes in the Hippocampus That May Have an Impact on Behavior after Chronic Social Stress
Source: NeuroSci. 2024 Jun 6;5(2):192–200. doi: 10.3390/neurosci5020014 (PMC11469743; doi:10.3390/neurosci5020014)
Supplement: Supplementary file 1 [file neurosci-05-00014-s001.zip › neurosci-2995165-supplementary.pdf]

Supplementary material

Table .S1. PCR Primer specification

Gene	Description	Primer sequence (5´-3´)	GenBank accession No.
IL-6	Interleukin 6	F: GCCAGAGTCCTTCAGAGA<br>R: CCTTAGCCACTCCTTCTGT	NM_031168.1
TNFα	Tumor necrosis factor	F: ACGTGGAACTGGCAGAAGAG R:<br>TGCCACAAGCAGGAATGAGA	NM_013693.2
iNOS	Inductible nitric oxide synthase	F: GGATCTTCCCAGGCAACCA<br>R: CAATCCACAACTCGCTCCAA	NM 010927.3
IDO	Indoleamine 2,3-dioxygenase	F: AAAGCAATCCCCACTGTATCCA<br>R: TGCCTTTTCCAATGCTTTCAG	BC049931.1
TDO	Tryptophan 2,3-dioxygenase	F: CGATTCCAGGTCCCTTTCCA<br>R: AGCCTTGGTGCCCAGCAT	
GAPDH	Glyceraldehyde-6-phosphate dehydrogenase	F: CGGCCGCATCTTCTTGTG<br>R: GTGACCAGGCGCCCAATAC	NM_008084.2
HPRT	Hypoxanthine-guanine phosphoribosyltransferase	F: TGGGAGGCCATCACATTGT<br>R: TCCAGCAGGTCAGCAAAGAAC	NM_013556.2
